# Translation and Initial Validation of the Chinese Version of the Action Research Arm Test in People with Stroke

**DOI:** 10.1155/2019/5416560

**Published:** 2019-01-21

**Authors:** Jiang-Li Zhao, Pei-Ming Chen, Wen-Feng Li, Rui-Hao Bian, Ming-Hui Ding, Hai Li, Qiang Lin, Zhi-Qin Xu, Yu-Rong Mao, Dong-Feng Huang

**Affiliations:** ^1^Department of Rehabilitation Medicine, The First Affiliated Hospital, Sun Yat-sen University, Guangzhou 510080, Guangdong Province, China; ^2^Department of Rehabilitation Sciences, The Hong Kong Polytechnic University, Hong Kong; ^3^Department of Rehabilitation Medicine, The Fifth Affiliated Hospital of Guangzhou Medical University, Guangzhou, China

## Abstract

**Purpose:**

This study aimed to translate the English version of the Action Research Arm Test (ARAT) into Chinese and to evaluate the initial validation of the Chinese version (C-ARAT) in patients with a first stroke.

**Methods:**

An expert group translated the original ARAT from English into Chinese using a forward-backward procedure. Forty-four patients (36 men and 8 women) aged 22–80 years with a first stroke were enrolled in this study. The participants were evaluated using 3 stroke-specific outcome measures: C-ARAT, the upper extremity section of the Fugl–Meyer assessment (UE-FMA), and the Wolf Motor Function Test (WMFT). Internal consistency was analysed using Cronbach's *α* coefficients and item-scale correlations. Concurrent validity was determined using Spearman's rank correlation coefficients. Floor and ceiling effects were considered to be present when more than 20% of patients fell outside the preliminarily set lower or upper boundary, respectively.

**Results:**

The C-ARAT items yielded excellent internal consistency, with a Cronbach's *α* of 0.98 (p < 0.001) and item-total correlations ranging from 0.727 to 0.948 (p < 0.001). The C-ARAT exhibited good-to-excellent correlations with the UE-FMA and WMFT functional ability (WMFT-FA) scores, with respective *ρ* values of 0.824 and 0.852 (p < 0.001), and an excellent negative correlation with the WMFT performance time (WMFT-time), with a *ρ* value of -0.940 (p < 0.001). The C-ARAT subscales generally exhibited good-to-excellent correlations with stroke-specific assessments, with *ρ* values ranging from 0.773 to 0.927 (p < 0.001). However, the gross subscale exhibited moderate-to-good correlations with the UE-FMA and WMFT-FA scores, with respective *ρ* values of 0.665 and 0.720 (p < 0.001). No significant floor effect was observed, and a significant ceiling effect was observed only on the WMFT-time.

**Conclusions:**

The C-ARAT demonstrated excellent internal consistency and good-to-excellent concurrent validity. This test could be used to evaluate upper extremity function in stroke patients without cognitive impairment.

## 1. Introduction

Stroke, a leading cause of disability in adults worldwide, can result in highly complex clinical situations [1]. Approximately 80% of stroke survivors regain their locomotor function [2]. Among hemiplegic stroke patients, however, approximately 30–66% present with a nonfunctional paretic arm at 6 months after stroke, whereas only 5–20% demonstrate a complete functional recovery [3]. Upper extremity (UE) paralysis often limits patients' daily living activities and may reduce their quality of life [4]. Accordingly, upper limb functional measurements are essential to improving clinical practice and evaluating the efficacy of rehabilitative interventions [5]. An appropriate outcome measure could improve the diagnostic efficacy and symptom quantification, assist with the planning and follow-up of rehabilitative interventions, and improve communication between clinicians [6]. Furthermore, the precise time course of a recovery of arm paresis depends on the selected outcome measure [7].

Although numerous upper limb measurement tools have been used in stroke rehabilitation studies, only 15 of them have been applied in more than 5% of studies [5]. One such tool, the Action Research Arm Test (ARAT), primarily concerns the International Classification of Functioning, Disability, and Health activity level and has been applied in approximately 17% of studies [5]. This 19-item observational measure is used to assess UE performance in people with stroke and includes 4 domains: grasping, gripping, pinching, and gross movement. The ARAT involves observations of arm and hand movement during the performance of a range of reaching and grasping tasks. This test is simple and easy to prepare and can be administered to patients at mean time intervals of approximately 8 minutes [8].

Several previous studies have proven the good psychometric properties of the ARAT [8–14]. Hsieh et al. reported strong correlations between the English version of the ARAT and the UE part of the motor assessment scale, arm subscore of the motricity index, and UE part of the modified motor assessment chart (Pearson's r = 0.96, 0.87, and 0.94, resp.) in people with stroke [8]. Yozbatiran et al. also demonstrated the excellent construct validity of the ARAT, which correlated strongly with the UE section of the Fugl–Meyer assessment (UE-FMA; r = 0.94) in chronic stroke patients with moderate right hemiparesis [14]. Nordin et al. suggested that the ARAT showed satisfied intrarater and interrater reliability for patients after stroke [10]. Lin et al. proved that the ARAT yielded sufficient validity, responsiveness, and reliability in participants with stroke, with satisfactory minimal detectable changes for assessing disability [11]. Accordingly, the ARAT has been widely used in clinical and research studies [15–17].

The original ARAT protocol and manual were translated into Swedish [10]. To the best of our knowledge, only 2 published Chinese studies [18, 19] had estimated the validity and reliability of the original ARAT, and no study had reported on a translation of the English version of the ARAT into Chinese. Accordingly, there was a significant clinical need for a translated Chinese version of the ARAT (C-ARAT). This study aimed to translate the original ARAT into Chinese and explore the internal consistency, concurrent validity, and floor and ceiling effects of this test in people with stroke.

## 2. Methods

### 2.1. Translation

The original ARAT was translated from English into Chinese using a forward-backward procedure. The forward procedure was performed by 2 native Chinese speakers who accurately translated the scale from English to Chinese according to the original scale. Next, the 2 translators resolved any discrepancies and synthesised the results based on their translations. The backward procedure was then performed by 2 native English speakers who were blind to the original English version. The 2 translators were neither aware nor informed of the concepts explored to avoid information bias and unexpected meanings in the translated questionnaire. The translated questionnaire was then reviewed by an expert committee comprising the principal investigator, the 4 translators, 2 experienced physiotherapists, 2 occupational therapists, and 2 rehabilitation physicians. The expert committee reviewed all versions of the questionnaire and developed what would be considered the final version of the questionnaire for field testing [20].

### 2.2. Subjects

According to a previous study [12], a sample size of 40 subjects with stroke is sufficient to determine the internal consistency and concurrent validity of the C-ARAT. This study included 44 inpatients with stroke in the Department of Rehabilitation Medicine of the First Affiliated Hospital, Sun Yat-sen University, China, between August 2014 and March 2018. The inclusion criteria were as follows: (1) the occurrence of a first stroke with unilateral hemiparetic lesions confirmed by magnetic resonance imaging or computed tomography; (2) an interval of >6 days after stroke; (3) age of 18–80 years; (4) Brunnstrom motor recovery stage II or higher; (5) Modified Ashworth Scale score ≤2; (6) ability to maintain a sitting position for >30 minutes; (7) no severe deficits in communication, memory, and understanding [Mini Mental State Examination score ≥22]; and (8) no additional medical, cardiovascular, or orthopaedic condition or significant UE peripheral neuropathy. The participants' demographic details and major comorbidity data were collected from medical records. The demographic information is shown in Table 1. This study was approved by the Human Subjects Ethics Subcommittee of the First Affiliated Hospital, Sun Yat-sen University, China. Informed written consent was obtained from all of the participants.

### 2.3. Procedure

Prior to collecting baseline data, an experienced physiotherapist with 9 years of clinical experience in stroke rehabilitation was trained to properly administer the C-ARAT, WMFT, and UE-FMA according to recent guidelines [14, 21]. We used a random drawing to randomise the order of the UE outcome measures, which were administered in a quiet room. The C-ARAT, WMFT, and UE-FMA were applied to patients recruited from the Department of Rehabilitation Medicine of the First Affiliated Hospital, Sun Yat-sen University, China (n = 44). A sufficient rest period was provided during the assessment to avoid the influence of fatigue on the results. The entire assessment took approximately 1–2 hours.

### 2.4. Outcome Measures

#### 2.4.1. ARAT

The ARAT was developed by Lyle [22] in 1981 as a performance test for evaluating UE function and dexterity after stroke. Hsieh reported that the English version of the ARAT was reliable (interrater reliability = 0.98) for the assessment of people with stroke [8]. Yozbatiran et al. [14] presented a standardized approach along with a detailed test manual, which was translated by our expert group into a Chinese version according to a standard forward and backward translation protocol, as described above. The ARAT includes 19 items applied according to a standardized test kit [10]. Each item is graded on a 4-point original scale [0, unable to complete any part of the task within 60 s; 1, partial performance of the task within 60 s; 2, completion of the task but with great difficulty or in an abnormally long time (5–60 s); or 3, normal performance of the task within 5 s] [14]. The ARAT is categorized into 4 subtests: grasping (6 items; 0–18 points), gripping (4 items; 0–12 points), pinching (6 items; 0–18 points), and gross movement (3 items; 0–9 points). UE function is assessed unilaterally, beginning with the unaffected upper extremity. The scores of each item are summed to calculate a total score for each side within a range of 0–57 points. Each subtest of the ARAT is arranged in a hierarchical order wherein the most difficult item is tested first, the easiest item is tested second, and the difficulty of the items increases gradually thereafter.

#### 2.4.2. Wolf Motor Function Test

The WMFT is a widely used laboratory-based evaluation designed to assess UE function (test-retest reliability = 0.90–0.95) [23] in people with stroke [24–26]. This test comprises 17 items, including 2 strength items and 15 timed task performance items. We used the 15 timed task performance items in this study. This division enables the WMFT to yield 2 scores: a functional ability (FA) score, which quantifies performance quality, and a timed score (TIME), which quantifies the performance speed (in seconds). The FA score rates movement quality on a 6-point ordinal scale ranging from 0 to 5, with higher scores indicating less impairment or activity limitation. A maximum time of 120 seconds was allotted for each task. The final time score is the mean time required to execute all timed tasks. The reliability and validity of the WMFT have been reported in both chronic and subacute populations of stroke patients [27–31]. The WMFT has been translated into Chinese and been proved with good validity and reliability [32].

#### 2.4.3. Upper Extremity Session of Fugl–Meyer Assessment

The UE-FMA, which has been used in 36% of studies, is the most commonly used measure [5] and has excellent interrater reliability and construct validity [33, 34]. The UE-FMA has frequently been used to measure UE motor impairment [1, 35–37] and has been reported to yield good intrarater and interrater reliability and construct validity [34, 38–45]. The UE-FMA comprises 33 items that are scored using a 3-point ordinal scale (0, cannot perform; 1, partially performed; 2, fully performed) to yield a maximum possible total score of 66. The UE-FMA already has Chinese version and widely been used [32].

#### 2.4.4. Modified Ashworth Scale

The muscle tone in the elbow, wrist, and finger flexors was assessed using the Modified Ashworth Scale (range: 0–4) [46]. Here, a score ≥1 indicates spasticity.

### 2.5. Statistical Analysis

#### 2.5.1. Participants

The demographic and clinical characteristics of the participants in this study (n = 44) were demonstrated using descriptive statistics.

#### 2.5.2. Internal Consistency

In order to evaluate the quality of the translated ARAT, internal consistency was used to test the agreement of each item. The internal consistency of the C-ARAT was assessed using Cronbach's *α* coefficients and item-total correlations. Cronbach's *α* values with corresponding confidence intervals (CI) were calculated to determine the internal consistency between the items of the C-ARAT. Here, *α* values of <0.5, 0.5 to <0.6, 0.6 to <0.7, 0.7 to <0.8, 0.8 to <0.9, and ≥0.9 indicated unacceptable, poor, questionable, acceptable, good, and excellent internal consistency, respectively [47]. The item-total correlations were analysed using Pearson's correlation coefficients.

#### 2.5.3. Validity

The concurrent validity of the C-ARAT was assessed by computing the correlations of the C-ARAT score with the WMFT and UE-FMA scores. As the C-ARAT, WMFT, and UE-FMA are ordinal scales, Spearman's rank correlation coefficient (*ρ*) was used to evaluate these correlations. Here, *ρ* values between 0 and 0.25, between 0.25 and 0.50, between 0.50 and 0.75, and >0.75 represented weak, fair, moderate-to-good, and good-to-excellent correlations, respectively [48].

#### 2.5.4. Floor and Ceiling Effects

Floor and ceiling effects were defined as the means percentages of subjects who scored beyond the lower and upper boundaries of the total score, respectively. The cut-off for the floor and ceiling effects was set at 5% of the total score [12]. Therefore, scores <3, <4, and <4 points in the C-ARAT, UE-FMA, and WMFT-FA, respectively, and WMFT-time scores ≥114 seconds were determined as a floor effect. Scores >54, >62, and >71 points on the C-ARAT, UE-FMA, and WMFT-FA, respectively, and WMFT-time scores ≤6 seconds were determined as a ceiling effect. Floor or ceiling effect >20% of the sample size was considered significant [11].

All of the statistical analyses were performed using SPSS version 20.0. All of the applied tests were 2-tailed. The level of significance was set at a p value <0.05.

## 3. Results

### 3.1. Demographics

Forty-four individuals with a first stroke (36 men, 8 women) were enrolled in this study. The median of the participants' age was 57.50 years (range: 22–80 years). The median poststroke duration was 3.00 months (range: 0.5–80.27 months). Thirty-three and 11 patients had ischemic and haemorrhagic stroke, respectively. The right side was affected in 48% of participants. Twenty individuals had hypoesthesia in their affected arms, including 1 case of combined sensory hypoesthesia, 15 cases of superficial sensation hypoesthesia, and 4 cases of combined superficial and deep sensory hypoesthesia. No patient presented with hemineglect. Fifteen individuals had mild speech impediments that did not affect communication. Details of the 44 participants were provided in Table 1.

The C-ARAT, UE-FMA, and WMFT performance scores were summarized in Table 2. The participants had a median total C-ARAT score of 31.50 (range: 3–57), with median grasping, gripping, pinching, and gross motor scores of 12.00, 7.50, 5.50, and 6.00, respectively. The median UE-FMA score was 51.00 (range: 19–66). The median WMFT FA total score was 47.00 (range: 6–74), and the median WMFT time was 11.49 seconds (range: 1.37–120.00 seconds).

### 3.2. Internal Consistency

The data of all 44 subjects were pooled to calculate the internal consistency. The C-ARAT items exhibited excellent internal consistency, with a Cronbach's *α* value of 0.98. (p < 0.001). The Pearson correlation coefficients of the item-total correlations ranged from 0.727 to 0.948. Details were provided in Table 3.

### 3.3. Concurrent Validity

**Figure 1:**
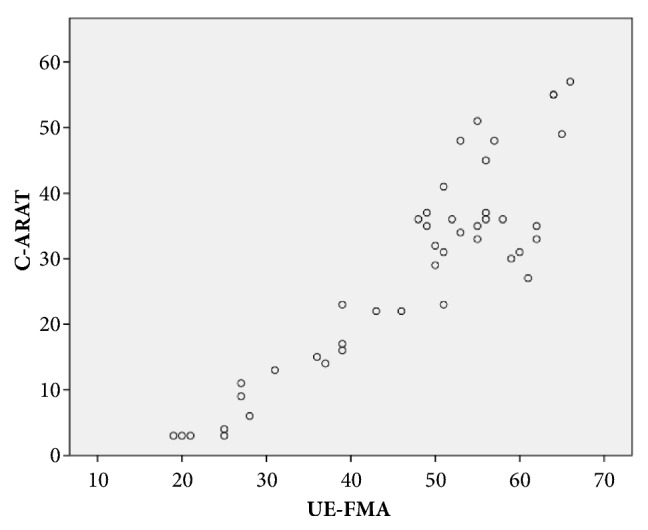
The relationship between the performance on the C-ARAT and UE-FMA.

The data on all 44 subjects were pooled to calculate the concurrent validity. The C-ARAT total score and UE-FMA score yielded a correlation of 0.824 (p < 0.001), indicating a good-to-excellent correlation. Most C-ARAT subscales also exhibited good-to-excellent correlations with the UE-FMA (grasping, *ρ* = 0.857; gripping, *ρ* = 0.844; pinching, *ρ* = 0.773; p < 0.001). However, the gross movement subscale exhibited a moderate-to-good correlation with the UE-FMA (*ρ* = 0.665; p < 0.001).  Figure 1 presented the relationship between the performance in the C-ARAT and that in the UE-FMA.

**Figure 2:**
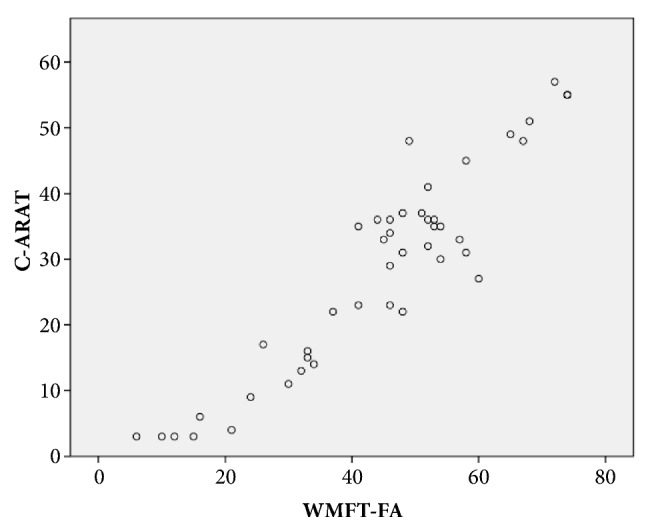
The relationship between the performance on the C-ARAT and WMFT-FA.

The C-ARAT total score and WMFT-FA score yielded a correlation coefficient of 0.852 (p < 0.001), which indicated a good-to-excellent correlation. Most of the C-ARAT subscales also exhibited good-to-excellent correlations with the WMFT-FA (grasping, *ρ* = 0.873; gripping, *ρ* = 0.917; pinching, *ρ* = 0.780; p < 0.001). The gross movement subscale exhibited a moderate-to-good correlation with the UE-FMA (*ρ* = 0.720; p < 0.001).  Figure 2 presented the relationship between the performances of the C-ARAT and WMFT-FA.

**Figure 3:**
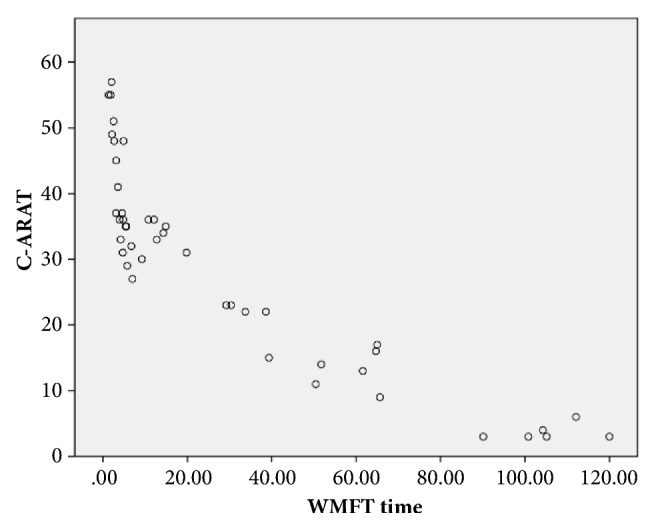
The relationship between the performance on the C-ARAT and WMFT time.

The C-ARAT scores and WMFT-time score yielded good-to-excellent correlations with *ρ* values >0.75; the respective *ρ* values for the total, grasping, gripping, pinching, and gross movement scores were -0.940 (p < 0.001), -0.894 (p < 0.001), -0.927 (p < 0.001), -0.903 (p < 0.001), and -0.782 (p < 0.001). In other words, the total C-ARAT and all subscales exhibited strong negative correlations with the WMFT-time.  Figure 3 presented the relationship between performance in the ARAT and WMFT-time.

Detailed results of the validity analyses were shown in Table 4.

### 3.4. Floor and Ceiling Effects

Table 5 demonstrated that the WMFT-time had a significant ceiling effect (40.9% of patients) but no floor effect. No significant floor or ceiling effects were observed in C-ARAT, UE-FMA, and WMFT-FA.

## 4. Discussion

Rapid increases in population aging and medical technique development have led to a growing number of people who suffer from stroke [49]. Furthermore, patients are gaining a greater awareness of the importance of quality of life, self-care, and daily living activities and are increasingly demanding the recovery of arm and hand function. However, approximately 85% of stroke survivors experience some degree of UE paresis [50], and it is difficult to assist these patients with the recovery of upper limb function as improper clinical decision was made. A reliable and valid assessment tool for evaluating UE function could help to improve the clinical decision making. This is the first study to translate the original ARAT protocol and manual into the Chinese language and to explore the concurrent validity, internal consistency, and floor and ceiling effects of this C-ARAT in a Chinese population of patients with first stroke.

In order to estimate how well the items measure the same concept in Chinese version of ARAT, the internal consistency was calculated. Internal consistency is one way to assess the quality of the translation. In this study, internal consistency is defined as agreement among all 19 items measuring the same traits of the construct and the subjects' performance [51]. Our results reported a satisfactory Cronbach's *α* coefficient of 0.98 for the C-ARAT, indicating excellent internal consistency. This result was consistent with previous studies [12, 52] which measured the internal consistency of original version of ARAT in people with subacute to chronic stroke. Meanwhile, the individual items of the C-ARAT demonstrated satisfactory item-total correlations (r > 0.727) [53], and Cronbach's *α* decreased by a maximum of only 0.002 if any single item was deleted. Therefore, each item of the C-ARAT was worthy of retention. Nijland et al. [12] also observed that the ARAT had excellent internal consistency (Cronbach's *α* = 0.98) in people with acute stroke. These findings showed that the C-ARAT showed high internal consistency as well as the original version, which may indicate that the Chinese version ARAT was well translated in each item.

Our results demonstrated a good-to-excellent correlation between the total scores of the C-ARAT and UE-FMA (Figure 1), in accordance with previous studies [11, 43, 45]. This indicated that the ARAT score may effectively assess not only function, but also motor impairment in the UE [8]. This study calculated a lower *ρ* value than those reported by Wei [43], See [45], and Lin [11]. In the study of chronic stroke survivors by Wei, higher *ρ* values of 0.93 and 0.92 were reported before and after training, respectively [43]. Compared with our study, Wei [43] included younger participants with a longer stroke onset. See evaluated 12 participants during 4 separate visits across the treatment period, for a total of 48 exams focused on validity, and reported a *ρ* value of 0.93 [45]. However, the sample size and exam procedure in our study differed from those used by See. In the study by Lin [11], participants with stroke were evaluated at 14, 30, 90, and 180 days after stroke, which corresponded to *ρ* values of 0.90, 0.90, 0.82, and 0.92, respectively. However, the poststroke duration and motor performance and intervention in that study differed considerably from those of our participants. Hsieh et al. [54] observed a moderate correlation (*ρ* = 0.71–0.74) between the UE-FMA and ARAT in chronic stroke survivors who received any 1 of 3 interventions (CIT, BAT, or conventional rehabilitation). The discrepant results between the study by Hsieh et al. and our study may be attributable to differences in severity of the motor impairment. The study by Hsieh et al. [54] included participants with mild functional disability in the proximal and distal UE (Brunnstrom stage 5). In contrast, the participants in our study had more severe disability on the affected side (Brunnstrom stage 3.6, proximal UE, and 4, distal UE).

Our results showed a strong correlation of the C-ARAT total score with the WMFT-FA score (Figure 2), which is consistent with the finding in previous studies [11, 12], especially that of Nijland et al. [12], which indicated that C-ARAT was well translated. A possible explanation was that the translation protocol was well prepared and the translators were professional. As a result, the original ARAT could be translated to Chinese version accurately. However, two studies [28, 54] reported results that differed from ours. The differences may be due to differences in the onset or motor performance before training. Our results also showed a significant negative correlation of the C-ARAT total score with the WMFT time score (Figure 3), in accordance with previous studies [12]. These strong correlations of the C-ARAT with the WMFT-FA and WMFT time suggest that C-ARAT has good concurrent validity with the gold standard measurement of motor function in stroke patients. These results support the validity of the C-ARAT as an outcome measure of UE function in stroke patients.

In addition, the C-ARAT subscales correlated strongly with stroke-specific assessments (*ρ* > 0.75) except for the gross movement subscale, which showed moderate-to-good correlations with the UE-FMA and WMFT-FA (*ρ* = 0.665–0.720). Nordin et al. [10] and Van der Lee et al. [55] reported that item 19 on the ARAT was problematic because of difficulties in distinguishing between categories 2 and 3. More studies are needed to further standardise the manual for item 19.

In our study, no significant floor and ceiling effect was observed in C-ARAT, UE-FMA, and WMFT-FA according to the criteria of floor and ceiling effect, which was consistent with the findings reported by Nijland [12]. A significant ceiling effect of WMFT-time was observed when compared to C-ARAT, which indicated that C-ARAT may be a more optimal assessment tool to evaluate the people with mild stroke when compared to WMFT-time. In addition, there was an interesting finding in our study. A slight floor effect was found in C-ARAT when compared to UE-FMA and WMFT-FA. This finding may indicate that C-ARAT was more sensitive to detect the clinical improvement in stroke survivors with mild-to-moderate motor impairment. One possible reason was that C-ARAT mainly assessed the motor performance of grip, grasp, pinch, and gross movement. Most of the assessment items correlated with the fine movement, which required the subjects to perform with a higher level of upper limb motor ability. In this way, as more than half of the assessment items in C-ARAT evaluate different aspects of the fine motor function, it showed a better discriminative ability in people with mild stroke. Comparatively, WMFT-time, WMFT-FA, and UE-FMA could be a more sensitive assessment tool in measuring the change of motor function in people with more severe impairment. By measuring the floor and ceiling effect of C-ARAT and some stroke-specific assessment tools, we found that these instruments may be useful in different impairment severity. However, precaution should be taken before making the conclusion. As only 44 subjects were included to analyse the floor and ceiling effect in our study, this finding may not be applied to the general stroke population. With more subjects included, we may draw a more reliable conclusion to determine the optimal assessment window for C-ARAT.

The C-ARAT, which is simple and rapid, assesses not only hand dexterity and strength but also the function of the whole UE. In contrast, the WMFT requires participants to change positions to sit on both sides and in front of the table, as well as a close facing position, and even demands that participants stand and lift a basket during the last task. As this test requires the ability to shift position, it is difficult for some stroke survivors, especially those in the early poststroke phase. In comparison, the ARAT does not require changes in body position and only requires the participant to sit in front of the table. Therefore, the ARAT is a satisfactory instrument for the assessment of UE function in stroke patients. Furthermore, the tools required for the ARAT are inexpensive and the assessment is less time-consuming than for other methods. Therefore, it is easily obtained and administered in clinical settings.

This study had some limitations. First, the sample size was small. Therefore, we could not conduct analyses according to the severity or type of stroke, type of intervention, or training time. The conclusion may only be applicable to the stroke survivors with the same severity. Second, the enrolled participants had a wide range of stroke onset times; some had been trained in grasping, gripping, or pinching exercises whereas others had no such training. These differences may have led to differences in performance during the C-ARAT. Finally, we only evaluated the concurrent validity, internal consistency, and floor and ceiling effects of the C-ARAT. Further research should explore the comprehensive psychometric characteristics, such as the intra- and interrater reliability, responsiveness, and predictive validity, of the instrument in stroke survivors at different stages.

## 5. Conclusion

In conclusion, the C-ARAT is an excellent and valid measure of UE function in stroke survivors. Our findings support the generalised clinical or research use of the C-ARAT in Chinese patients with stroke. However, further research is needed to evaluate the comprehensive psychometric characteristics of the C-ARAT.

## Figures and Tables

**Table 1:** Characteristics of the study participants (n=44).

Variable	Study sample n=44
	Values
Age (years)	57.50 (22-80)
Onset (months)	3.00 (0.50-80.27)
Mini mental state examination	27 (22-30)
Sex	
Male	36 (82)
Female	8 (18)
Stroke type	
Ischemic	33 (75)
Hemorrhagic	11 (25)
Affected side	
Right	21 (48)
Left	23 (52)
Dominance	
Right	44 (100)
Dominant side affected	21 (48)
Sensory disorder UE	20 (45.5)
Mild problem on speech	15 (34.1)
Brunnstrom stage	
Proximal UE	4 (2-6)
Distal UE	4 (2-6)

*Note*. Values are median (range) or n (%).

**Table 2:** Scores on outcome measures.

Variable	Median (range)
C-ARAT scores	
Total score	31.50 (3-57)
Grasp score	12.00 (0-18)
Grip score	7.50 (0-12)
Pinch score	5.50 (0-18)
Gross motor score	6.00 (3-9)
UE-FMA score	51.00 (19-66)
WMFT scores	
Function score	47.00 (6-74)
Time score (s)	11.49 (1.37-120.00)

C-ARAT, Chinese version of Action Research Arm Test; UE-FMA, Upper-Extremity subscale of the Fugl-Meyer Assessment; WMFT, Wolf Motor Function Test.

**Table 3:** Internal consistency of the C-ARAT.

Item	Item-total correlation	Alpha if item deleted
*Grasp*		
(1) Block 10 cm	0.897	0.978
(2) Block 2.5 cm	0.948	0.978
(3) Block 5 cm	0.929	0.978
(4) Block 7.5 cm	0.899	0.978
(5) Cricket ball	0.879	0.978
(6) Sharpening stone	0.921	0.978
*Grip*		
(7) Pour water from glass to glass	0.877	0.978
(8) Tube 2.25 cm	0.896	0.978
(9) Tube 1 cm	0.848	0.979
(10) Put washer over a bolt	0.846	0.979
*Pinch*		
(11) Ball 6 mm 3^rd^ finger and thumb	0.727	0.98
(12) Marble 1^st^ finger and thumb	0.924	0.978
(13) Ball 6 mm 2^nd^ finger and thumb	0.783	0.98
(14) Ball 6 mm 1^st^ finger and thumb	0.879	0.979
(15) Marble 3^rd^ finger and thumb	0.874	0.979
(16) Marble 2^nd^ finger and thumb	0.893	0.978
*Gross movements*		
(17) Hand behind head	0.790	0.98
(18) Hand on top of head	0.777	0.98
(19) Hand to mouth	0.824	0.979

Note: Cronbach's *α* coefficient for the entire C-ARAT equals 0.98.

**Table 4:** Spearman's correlation coefficient (*ρ*) between C-ARAT scores and those of UE-FMA, WMFT-FA, and WMFT-Time.

Variable	UE-FMA	WMFT-FA	WMFT-Time
C-ARAT Total score	0.824^a^*∗∗*	0.852^a^*∗∗*	-0.940^a^*∗∗*
C-ARAT Grasp score	0.857^a^*∗∗*	0.873^a^*∗∗*	-0.894^a^*∗∗*
C-ARAT Grip score	0.844^a^*∗∗*	0.917^a^*∗∗*	-0.927^a^*∗∗*
C-ARAT Pinch score	0.773^a^*∗∗*	0.780^a^*∗∗*	-0.903^a^*∗∗*
C-ARAT Gross score	0.665^b^*∗∗*	0.720^b^*∗∗*	-0.782^a^*∗∗*

*Note*. *ρ* values indicate correlation coefficients by Spearman's rank correlation coefficient.

C-ARAT, Chinese version of Action Research Arm Test; UE-FMA, Upper-Extremity subscale of the Fugl-Meyer Assessment; WMFT, Wolf Motor Function Test; WMFT-FA, Wolf Motor Function Test functional ability

^a^Excellent correlation.

^b^Moderate correlation

p<0.05 indicates significant correlations.

*∗∗*p<0.001.

**Table 5:** Floor and ceiling effects of the 3 measures.

	C-ARAT	UE-FMA	WMFT-FA	WMFT-Time
Floor Effect	0 (0)	0 (0)	0 (0)	1 (2.3)
Ceiling Effect	3 (6.8)	4 (9.1)	3 (6.8)	18 (40.9)

*Note*. Values are n (%).

## Data Availability

The ordinal data used to support the findings of this study are available from the corresponding author upon request.
